# Supplementary material for: The Role of Mitochondria in Obstructive Sleep Apnea: Implications for the Upper Airway Muscles
Source: Int J Mol Sci. 2025 Oct 30;26(21):10562. doi: 10.3390/ijms262110562 (PMC12610555; doi:10.3390/ijms262110562)
Supplement: Supplementary file 1 [file ijms-26-10562-s001.zip › ijms-3823545-supplementary.pdf]

Category	Species subset	Search string (PubMed)	Applied filters	Number of records retrieved	Notes
**Muscle and mitochondrial structure**	Human	("obstructive sleep apnea"[tiab] OR "sleep apnoea"[tiab] OR "sleep-disordered breathing"[tiab] OR snor*[tiab]) AND (muscle[tiab]) AND ("upper airway"[tiab] OR geniogloss*[tiab] OR palatopharyngeal[tiab] OR "soft palate"[tiab] OR uvula[tiab]) AND (histology[tiab] OR morphology[tiab] OR histochem*[tiab] OR immunohistochemistry[tiab] OR "fiber type"[tiab] OR biopsy[tiab] OR myopath*[tiab] OR ultrastructur*[tiab] OR mitochondria*[tiab] OR electromyograph*[tiab] OR EMG[tiab] OR oxidative[tiab] OR neurogenic[tiab]) NOT review[pt] AND "humans"[MeSH Terms]	English	163	
**Muscle and mitochondrial structure**	Experimental	("obstructive sleep apnea"[tiab] OR "hypoxia"[tiab] OR "intermittent hypoxia"[tiab] OR "chronic intermittent hypoxia"[tiab] OR "chronic hypoxia"[tiab] OR "sustained hypoxia"[tiab] OR "hypobaric hypoxia"[tiab] OR "episodic hypoxia"[tiab] OR "hypercapnic hypoxia"[tiab]) AND (("upper airway"[tiab] OR genioglossal[tiab] OR genioglossus[tiab] OR "pharyngeal dilator"[tiab] OR sternohyoid[tiab] OR "upper airway dilator"[tiab]) AND muscle[tiab]) AND (morpholog*[tiab] OR histolog*[tiab] OR histochem*[tiab] OR ultrastructur*[tw] OR mitochondria*[tiab] OR oxidative[tiab] OR contractile[tiab] OR "fiber type"[tiab] OR myopath*[tiab]) AND (rat[tiab] OR rats[tiab] OR mouse[tiab] OR mice[tiab] OR murine[tiab] OR "animal model"[tiab] OR "in vitro"[tiab] OR "cell culture"[tiab]) NOT review[pt]	English	53	

**Mitochondrial function**	Human	("obstructive sleep apnea"[tiab] OR "sleep apnea hypopnea"[tiab] OR snorer*[tiab] OR snoring[tiab]) AND (muscle[tw]) AND ("upper airway"[tiab] OR genioglossus[tiab] OR genioglossal[tiab] OR sternohyoid[tiab] OR "pharyngeal dilator"[tiab] OR "upper airway dilator"[tiab] OR tongue[tiab]) AND (mitochondria*[tiab] OR oxidative[tiab] OR "oxidative stress"[tiab] OR ROS[tw] OR "electron transport"[tiab] OR OXPHOS[tiab] OR "respiration"[tiab] OR "respiratory chain"[tiab] OR "cytochrome c oxidase"[tiab] OR metabolic[tw] OR proteomic[tiab] OR proteomics[tiab] OR antioxidant*[tw] OR histochem*[tiab]) AND "humans"[MeSH Terms] NOT review[pt]	English	34	
**Mitochondrial function**	Experimental	("obstructive sleep apnea"[tiab] OR "intermittent hypoxia"[tiab] OR "chronic intermittent hypoxia"[tiab] OR "chronic hypoxia"[tiab] OR hypoxia[tiab]) AND ("upper airway"[tiab] OR genioglossus[tiab] OR genioglossal[tiab] OR sternohyoid[tiab] OR "pharyngeal dilator"[tiab] OR "upper airway dilator"[tiab] OR tongue[tiab]) AND (mitochondria*[tiab] OR oxidative[tiab] OR "oxidative stress"[tiab] OR ROS[tw] OR "electron transport"[tiab] OR OXPHOS[tiab] OR "respiration"[tiab] OR "respiratory chain"[tiab] OR "cytochrome c oxidase"[tiab] OR metabolic[tw] OR proteomic[tiab] OR proteomics[tiab] OR antioxidant*[tw]) AND (rat[tiab] OR rats[tiab] OR mouse[tiab] OR mice[tiab] OR murine[tiab] OR "animal model"[tiab] OR "in vivo"[tiab] OR "in vitro"[tiab] OR "cell culture"[tiab] OR "muscle satellite cell*"[tiab]) NOT review[pt]	English	84	

**HIF-1–induced regulators (subset of function)**	Human	("obstructive sleep apnea"[tiab] OR "obstructive sleep apnoea"[tiab] OR "sleep apnea hypopnea"[tiab] OR "sleep apnoea hypopnoea"[tiab] OR snorer*[tiab] OR snoring[tiab]) AND (muscle[tw]) AND ("upper airway"[tiab] OR genioglossus[tiab] OR genioglossal[tiab] OR sternohyoid[tiab] OR "pharyngeal dilator"[tiab] OR "upper airway dilator"[tiab] OR tongue[tiab]) AND (mitochondria*[tiab] OR "mitochondrial function"[tiab] OR "mitochondrial respiration"[tiab] OR "respiratory chain"[tiab] OR "oxidative phosphorylation"[tiab] OR "electron transport"[tiab] OR "cytochrome c oxidase"[tiab] OR OXPHOS[tiab] OR ROS[tw] OR oxidative[tiab] OR "oxidative stress"[tiab] OR antioxidant*[tw] OR metabolic[tw] OR proteomic[tiab] OR proteomics[tiab] OR histochem*[tiab]) AND (PDK[tiab] OR "pyruvate dehydrogenase kinase"[tiab] OR LDHA[tiab] OR "lactate dehydrogenase A"[tiab] OR COX4-2[tiab] OR "cytochrome c oxidase subunit 4 isoform 2"[tiab] OR LON[tiab] OR "Lon protease"[tiab] OR HIGD1A[tiab] OR HIGD-1A[tiab] OR "hypoxia inducible gene domain 1A"[tiab] OR NDUFA4L2[tiab] OR "NADH dehydrogenase (ubiquinone) 1 alpha subcomplex 4-like 2"[tiab] OR TMEM26B[tiab] OR "transmembrane protein 26B"[tiab] OR ISCU1[tiab] OR ISCU2[tiab] OR "iron-sulfur cluster scaffold protein"[tiab] OR NDUFA4[tiab] OR SDHD[tiab] OR "succinate dehydrogenase subunit D"[tiab] OR COX10[tiab] OR DRP1[tiab] OR "dynamin-related protein 1"[tiab] OR FUNDC1[tiab] OR OPA1[tiab] OR "optic atrophy 1"[tiab] OR BNIP3[tiab] OR SIAH2[tiab] OR "seven in absentia homolog 2"[tiab] OR "miR-210"[tiab] OR "microRNA-210"[tiab] OR "miR210"[tiab] OR "miR-137"[tiab] OR "microRNA-137"[tiab] OR "miR137"[tiab] OR "miR-17-5p"[tiab] OR "microRNA-17-5p"[tiab] OR "miR17-5p"[tiab]) AND "humans"[MeSH Terms] NOT (rats[tw] OR mice[tw] OR murine[tw] OR rabbit[tw]) NOT review[pt] NOT "case reports"[pt]	English	1	Focused on HIF-1 Induced regulators of mitochondrial function

**HIF-1–induced regulators (subset of function)**	Experimental	("obstructive sleep apnea"[tiab] OR "obstructive sleep apnoea"[tiab] OR "intermittent hypoxia"[tiab] OR "chronic intermittent hypoxia"[tiab] OR "chronic hypoxia"[tiab] OR hypoxia[tiab]) AND ("upper airway"[tiab] OR genioglossus[tiab] OR genioglossal[tiab] OR sternohyoid[tiab] OR "pharyngeal dilator"[tiab] OR "upper airway dilator"[tiab] OR tongue[tiab]) AND (mitochondria*[tiab] OR "mitochondrial function"[tiab] OR "mitochondrial respiration"[tiab] OR "respiratory chain"[tiab] OR "oxidative phosphorylation"[tiab] OR OXPHOS[tiab] OR ROS[tw] OR oxidative[tiab] OR "oxidative stress"[tiab] OR antioxidant*[tw] OR metabolic[tw]) AND (PDK[tiab] OR "pyruvate dehydrogenase kinase"[tiab] OR LDHA[tiab] OR "lactate dehydrogenase A"[tiab] OR COX4-2[tiab] OR "cytochrome c oxidase subunit 4 isoform 2"[tiab] OR LON[tiab] OR "Lon protease"[tiab] OR HIGD1A[tiab] OR "hypoxia inducible gene domain 1A"[tiab] OR NDUFA4L2[tiab] OR "NADH dehydrogenase (ubiquinone) 1 alpha subcomplex 4-like 2"[tiab] OR TMEM26B[tiab] OR "transmembrane protein 26B"[tiab] OR ISCU1[tiab] OR ISCU2[tiab] OR "iron-sulfur cluster scaffold protein"[tiab] OR NDUFA4[tiab] OR SDHD[tiab] OR "succinate dehydrogenase subunit D"[tiab] OR COX10[tiab] OR DRP1[tiab] OR "dynamin-related protein 1"[tiab] OR FUNDC1[tiab] OR OPA1[tiab] OR "optic atrophy 1"[tiab] OR BNIP3[tiab] OR SIAH2[tiab] OR "seven in absentia homolog 2"[tiab] OR "miR-210"[tiab] OR "microRNA-210"[tiab] OR "miR-137"[tiab] OR "microRNA-137"[tiab] OR "miR-17-5p"[tiab] OR "microRNA-17-5p"[tiab]) AND (rat[tiab] OR rats[tiab] OR mouse[tiab] OR mice[tiab] OR murine[tiab] OR rabbit[tiab] OR "animal model"[tiab] OR "in vivo"[tiab] OR "in vitro"[tiab] OR "cell culture"[tiab] OR "muscle satellite cell*"[tiab]) NOT review[pt] NOT "case reports"[pt]	English	3	

**Mitochondrial quality control**	Human	("obstructive sleep apnea"[tiab] OR "sleep apnea hypopnea"[tiab] OR snorer*[tiab] OR snoring[tiab]) AND (muscle[tw] OR genioglossus[tiab] OR genioglossal[tiab] OR "musculus uvulae"[tiab] OR palate[tiab] OR "pharyngeal muscle"[tiab] OR "palate muscle"[tiab] OR uvula*[tiab] OR palatopharyngeal[tiab] OR "soft palate"[tiab] OR tongue[tiab]) AND ("mitochondrial dynamics"[tiab] OR mitophagy[tiab] OR "mitochondrial fission"[tiab] OR "mitochondrial fusion"[tiab] OR "mitochondrial biogenesis"[tiab] OR "PGC-1α"[tiab] OR "PGC1α"[tiab] OR "PINK1"[tiab] OR "Parkin"[tiab] OR "Drp1"[tiab] OR "Mfn1"[tiab] OR "Mfn2"[tiab] OR "Opa1"[tiab] OR "mitochondrial turnover"[tiab] OR "quality control"[tiab] OR (("mitochondrial DNA"[tiab] OR "mtDNA"[tiab]) AND "copy number"[tiab])) AND "humans"[MeSH Terms] NOT review[pt]	English	4	
**Mitochondrial quality control**	Experimental	("obstructive sleep apnea"[tiab] OR "intermittent hypoxia"[tiab] OR "chronic intermittent hypoxia"[tiab] OR "chronic hypoxia"[tiab] OR "sustained hypoxia"[tiab] OR "hypobaric hypoxia"[tiab] OR "episodic hypoxia"[tiab] OR "hypercapnic hypoxia"[tiab]) AND (("upper airway"[tiab] OR genioglossal[tiab] OR genioglossus[tiab] OR "pharyngeal dilator"[tiab] OR sternohyoid[tiab] OR "upper airway dilator"[tiab] OR ("pharyngeal"[tiab] AND muscle[tiab]) OR "dilator muscle"[tiab]) AND muscle[tiab]) AND ("mitochondrial dynamics"[tiab] OR mitophagy[tiab] OR "mitochondrial fission"[tiab] OR "mitochondrial fusion"[tiab] OR "mitochondrial biogenesis"[tiab] OR "PGC-1α"[tiab] OR "PGC1α"[tiab] OR "PINK1"[tiab] OR "Parkin"[tiab] OR "Drp1"[tiab] OR "Mfn1"[tiab] OR "Mfn2"[tiab] OR "Opa1"[tiab] OR "mitochondrial turnover"[tiab] OR "quality control"[tiab] OR (("mtDNA"[tiab] OR "mitochondrial DNA"[tiab]) AND "copy number"[tiab])) AND (rat[tiab] OR rats[tiab] OR mouse[tiab] OR mice[tiab] OR murine[tiab] OR "animal model"[tiab] OR "in vitro"[tiab] OR "cell culture"[tiab] OR "muscle satellite cells"[tiab])	English	5	

**Oxidative stress**	Human	(("oxidative stress"[ti] OR ("oxidative stress"[ti] AND ("evaluation"[tiab] OR "assessment"[tiab] OR "analysis"[tiab] OR "role"[tiab] OR "biomarkers"[tiab] OR "marker"[tiab] OR "markers"[tiab])))) AND ("obstructive sleep apnea"[tiab] OR "obstructive sleep apnea syndrome"[tiab] OR OSA[tiab] OR OSAHS[tiab]) AND (human[mh] OR humans[mh] OR "patients"[tiab] OR "subjects"[tiab] OR "clinical study"[tiab] OR "clinical trial"[tiab] OR "cohort"[tiab] OR "cross-sectional"[tiab] OR "case-control"[tiab]) NOT review[pt] NOT "case reports"[pt] NOT (rat[tw] OR rats[tw] OR mouse[tw] OR mice[tw] OR murine[tw] OR rabbit[tw])	English + Humans	93	Among the retrieved articles, we additionally selected those investigating oxidative stress biomarkers in biological fluids, based on the presence of all the following criteria: (1) quantification of specific molecular oxidative biomarkers; (2) assessment of the association with OSA severity or its therapeutic modulation; (3) measurement performed in blood or other non-invasive biological fluids; and (4) primary biochemical investigation of oxidative stress

**Oxidative stress**	Experimental	("obstructive sleep apnea"[tiab] OR "intermittent hypoxia"[tiab] OR "chronic intermittent hypoxia"[tiab] OR "chronic hypoxia"[tiab] OR "sustained hypoxia"[tiab] OR "hypobaric hypoxia"[tiab] OR "episodic hypoxia"[tiab] OR "hypercapnic hypoxia"[tiab]) AND (("upper airway"[tiab] AND muscle[tiab]) OR genioglossal[tiab] OR genioglossus[tiab] OR "pharyngeal dilator"[tiab] OR sternohyoid[tiab] OR "upper airway dilator"[tiab] OR ("pharyngeal"[tiab] AND muscle[tiab]) OR "dilator muscle"[tiab]) AND ("oxidative stress"[tw] OR "reactive oxygen species"[tiab] OR ROS[tw] OR antioxidant[tiab] OR antioxidants[tw] OR "redox imbalance"[tiab] OR "lipid peroxidation"[tiab] OR "superoxide dismutase"[tiab] OR catalase[tiab] OR glutathione[tiab]) AND (rat[tiab] OR rats[tiab] OR mouse[tiab] OR mice[tiab] OR murine[tiab] OR "animal model"[tiab] OR "in vitro"[tiab] OR "cell culture"[tiab] OR "muscle satellite cells"[tiab] OR rodent*[tiab] OR rabbit*[tiab] OR Wistar[tiab] OR "Sprague-Dawley"[tiab] OR Zucker[tiab] OR animals[mh] OR "models, animal"[mh]) NOT review[pt]	English	23	
